# High-Throughput Analysis of Synthetic Peptides for the Immunodiagnosis of Canine Visceral Leishmaniasis

**DOI:** 10.1371/journal.pntd.0001310

**Published:** 2011-09-13

**Authors:** Angélica R. Faria, Míriam M. Costa, Mário S. Giusta, Gabriel Grimaldi, Marcus L. O. Penido, Ricardo T. Gazzinelli, Hélida M. Andrade

**Affiliations:** 1 Departamento de Parasitologia, Instituto de Ciências Biológicas, Universidade Federal de Minas Gerais, Belo Horizonte, Brasil; 2 Departamento de Bioqu$mica e Imunologia, Instituto de Ciências Biológicas, Universidade Federal de Minas Gerais, Belo Horizonte, Brasil; 3 Instituto Oswaldo Cruz – Fundação Oswaldo Cruz, Rio de Janeiro, Brasil; 4 Centro de Pesquisas René Rachou – Fundação Oswaldo Cruz, Belo Horizonte, Brasil; 5 Division of Infectious Diseases and Immunology, University of Massachusetts Medical School, Worcester, Massachusetts, United States of America; New York University School of Medicine, United States of America

## Abstract

**Background:**

Visceral leishmaniasis is the most severe form of leishmaniasis. Approximately 20% of zoonotic human visceral leishmaniasis worldwide is caused by *Leishmania infantum*, which is also known as *Leishmania chagasi* in Latin America, and disease incidence is increasing in urban and peri-urban areas of the tropics. In this form of disease, dogs are the main reservoirs. Diagnostic methods used to identify *Leishmania* infected animals are not able to detect all of the infected ones, which can compromise the effectiveness of disease control. Therefore, to contribute to the improvement of diagnostic methods for canine visceral leishmaniasis (CVL), we aimed to identify and test novel antigens using high-throughput analysis.

**Methodology/Principal Findings:**

Immunodominant proteins from *L. infantum* were mapped in silico to predict B cell epitopes, and the 360 predicted peptides were synthesized on cellulose membranes. Immunoassays were used to select the most reactive peptides, which were then investigated with canine sera. Next, the 10 most reactive peptides were synthesized using solid phase peptide synthesis protocol and tested using ELISA. The sensitivity and specificity of these peptides were also compared to the EIE-LVC Bio-Manguinhos kit, which is recommended by the Brazilian Ministry of Health for use in leishmaniasis control programs. The sensitivity and specificity of the selected synthesized peptides was as high as 88.70% and 95.00%, respectively, whereas the EIE-LVC kit had a sensitivity of 13.08% and 100.00% of specificity. Although the tests based on synthetic peptides were able to diagnose up to 94.80% of asymptomatic dogs with leishmaniasis, the EIE-LVC kit failed to detect the disease in any of the infected asymptomatic dogs.

**Conclusions/Significance:**

Our study shows that ELISA using synthetic peptides is a technique with great potential for diagnosing CVL; furthermore, the use of these peptides in other diagnostic methodologies, such as immunochromatographic tests, could be beneficial to CVL control programs.

## Author Summary

Globally, the number of new human cases of visceral leishmaniasis (VL) is estimated to be approximately 500,000 per year. This is the most severe of all forms of leishmaniasis, and the zoonotic form of VL, caused by *Leishmania infantum (*also known as *Leishmania chagasi*), represents 20% of human visceral leishmaniasis worldwide; additionally, its prevalence is increasing in urban and peri-urban areas of the tropics. In Brazil, the identification and elimination of infected dogs, which act as a reservoir for *Leishmania* parasites, is a control measure employed in addition to the use of insecticides against the vectors and the identification and treatment of infected humans. Currently, the diagnostic methods employed to identify infected animals are not able to detect all of these dogs, which compromises the effectiveness of control measures. Moreover, one of the most important issues in controlling VL is the difficulty of diagnosing asymptomatic dogs, which act as parasite reservoirs. Therefore, to contribute to the improvement of the diagnostic methods for CVL, we aimed to identify and characterize new antigens that were more sensitive and specific and could be applied in epidemiologic surveys.

## Introduction

Leishmaniasis, which is one of the major parasitic diseases recognized by the World Health Organization, affects approximately 1–2 million individuals annually. Dogs are considered the main domestic reservoir of *Leishmania infantum* (also known as *L. chagasi*) [1], which is the causative agent of zoonotic visceral leishmaniasis (VL) in both the Old and New Worlds [2]. In endemic areas, up to 85% of infected dogs may be asymptomatic [3], and they serve as reservoir for vector transmission to susceptible animals and humans [4].

The epidemiological control of VL in Brazil involves the elimination of infected dogs, widespread insecticide use and the systematic treatment of human cases [5]. Reliable diagnostic tests for *L. infantum* detection are essential to prevent disease transmission and the unnecessary culling of dogs. Given the frequency of asymptomatic infections in dogs and the difficulty of direct parasite detection, the development of rapid and accurate indirect diagnostic methods for canine infection is essential for VL surveillance programs. The principal serodiagnostic tests include the immunofluorescent antibody test (IFAT) and the enzyme-linked immunosorbent assay (ELISA). These conventional tests employ crude antigen preparations of either whole promastigotes or their soluble extracts, which limits assay standardization and result reproducibility [6].

An alternative method for the production of antigens for immunoassays is the synthesis of peptides. These peptides are relatively simple to synthesize and are cheaper to produce compared to the production of whole proteins [7]. In general, the use of synthetic peptides increases the specificity of immunoassays compared to crude antigens [8].

In previous studies, we identified almost 50 immunodominant proteins of *L. infantum,* mapped their B cell epitopes and submitted 180 peptides to Spot synthesis and immunoassay. A total of 25 peptides showed promising characteristics for serodiagnosis of visceral leishmaniasis [9]. Here, we increased the B cell epitopes mapping, performed a high-throughput analysis of 360 peptides and selected the top 10 peptides for ELISA testing. When assessed, the specificity and sensitivity of the selected peptides was as high as 88.7% and 95.0%, respectively. These new antigens represent solid candidate peptides for the diagnosis of VL with great accuracy, especially in asymptomatic animals.

## Methods

### Ethics Statement

Experiments with dogs were performed in compliance with the guidelines of the Institutional Animal Care and Committee on Ethics of Animal Experimentation (Comitê de Ética em Experimentação Animal – CETEA, national guidelines Lei 11.794, de 8 de outubro de 2008) from Universidade Federal de Minas Gerais (UFMG); protocol 211/07 was approved on 03/12/2008.

### Canine Sera

For the initial screening of *Leishmania* antigens on cellulose membranes, we used a pool of sera from ten animals per experimental group, *i.e.*, chronically infected dogs and uninfected control dogs. The chronically infected dogs were naturally infected with *Leishmania,* and they were found in the metropolitan region of Belo Horizonte, Minas Gerais state, Brazil, rescued and maintained in our facility for laboratory and clinical evaluations. VL in chronically infected dogs was certified by the presence of clinical symptoms and parasitological tests that were conducted on bone marrow cells examined by optical microscopy. The uninfected dogs were negative based on parasitological as well as serological tests for VL; these animals served as negative controls in our study. Blood from all the dogs was withdrawn and maintained at room temperature for 3 h to obtain serum. For each animal in a group, 100 µL of serum was deposited in a single tube to obtain a pool of sera that was representative of chronically infected or uninfected dogs; each group was comprised of 10 dogs.

For the ELISA tests, we used the serum described above and 62 serum samples from 23 symptomatic and 39 asymptomatic dogs from Pancas, Espírito Santo state, Brazil. Dogs were scored for 6 typical signs of canine visceral leishmaniasis: alopecia, dermatitis, chancres, conjunctivitis, onychogryphosis and lymphadenopathy. Each sign was scored on a semi-quantitative scale from 0 (absent) to 3 (severe), and these scores were added together to give an overall clinical score. Dogs with a total score of 0-2 were arbitrarily classed as asymptomatic, 3-6 as oligosymptomatic and 7-18 as symptomatic [10]. Additionally, several serum samples from dogs that were experimentally infected with other pathologies were also tested; samples from dogs that were seropositive for *Trypanosoma cruzi* by RIFI (n = 15) and positive for *Leishmania braziliensis* based on parasitological examination and molecular identification (n = 20) were kindly provided by Prof Dr. Ricardo Toshio Fujiwara of UFMG and Prof Dr. Alexandre Barbosa Reis of UFOP, respectively.

### Epitope Identification and Spot Synthesis

Previously, we identified almost 50 immunodominant proteins from *L. infantum,* performed the mapping of their B cell epitopes using BepiPred program that is based on propensity scale methods (http://www.cbs.dtu.dk/services/BepiPred/ [11]), and a total of 180 peptides were submitted for Spot synthesis and immunoassays, and 25 peptides were shown to be of interest for use in VL serodiagnosis [9]. Here, we completed the mapping of the same proteins [9] using two different programs: ABCPred, based on machine learning methods that apply a recurrent neural network (http://www.imtech.res.in/raghava/abcpred [12]) and BCPreds, which is also based on machine learning methods but involve those that apply a support vector machine (http://ailab.cs.iastate.edu/bcpreds/ [13]). Epitopes that were predicted by the two programs simultaneously (excluding those previously identified [9]) were synthesized using the Spot synthesis method [14] on derivatized cellulose membranes with an Ala-Ala linker; peptide size ranged from 9 to 14 amino acids [15].

### Immunoassays with cellulose-bound peptides

Initially, the selection of the most immunoreactive peptides was performed using immunoassays of pooled canine serum (previously described in [9]) and alkaline phosphatase-conjugated goat anti-dog immunoglobulin G. We tested all of the peptides mapped in both studies. The relative intensity of the spots representing each peptide was determined by overlapping positive and negative membranes with ImageMaster™ Platinum program. Peptides with relative intensity values of 2 or greater (RI ≥ 2) were considered potential candidate antigens [16]. Next, new membranes were synthesized with only the selected peptides, and they were tested with individual canine sera. To evaluate these assays, cut off values were calculated for each peptide using the mean color intensity + 2 standard deviations (SD) from 5 known negative individual canine sera. All assays were performed in duplicate.

### Synthesis of soluble peptides

Based on the results of immunoassays conducted using cellulose-bound peptides that were probed with canine sera, 10 peptides that exhibited reactivity with the largest number of serum samples from infected animals were chemically synthesized using 9-florenyl-methoxy-carbonyl (Fmoc) chemistry [17] in an automated synthesizer, model PSSM8 (Shimadzu, Kyoto, Japan). Peptide purity was assessed with reverse-phase high performance liquid chromatography (HPLC) and mass spectrometry (MALDI-TOF-TOFAutoFlexIII™, BrukerDaltonics, Billerica, Massachussets, USA). The synthetic peptides were diluted in PBS and used as antigens in ELISA assays.

### ELISA

All ELISA procedures were optimized in terms of antigen concentrations and the dilutions of serum and conjugated immunoglobulins to develop a reproducible and robust assay. The optimal antigen concentration was 20µg/mL. A clear separation between sera from *L. infantum*-infected and uninfected animals was possible using 1∶100 dilutions for sera and 1∶5,000 dilutions for the conjugated immunoglobulins.

Falcon flexible microtitration plates purchased from Becton Dickinson Labware Europe (Becton Dickinson, France S.A.) were coated for 16 h approximately with 100 µL/well of synthetic peptides (20 µg/mL) in PBS. Wells were then blocked with 5% powdered skim milk in PBS/T (PBS containing 0.05% Tween20) at 37°C for 1 hour. Serum samples, diluted 1∶100 in PBS/T containing 0.5% powdered skim milk, were added and incubated at 37°C for 1 hour. Plates were washed three times with PBS/T and then incubated with peroxidase-conjugated anti-dog immunoglobulin G (Sigma-Aldrich, St. Louis, MO) diluted 1∶5,000 in PBS/T containing 0.5% powdered skim milk at 37°C for 1 h. After washing three times with PBS/T, reactions were developed with Fast-OPD™ (Sigma-Aldrich, St. Louis, MO). Plates were incubated for 30 min in the dark. The reactions were stopped with 2 M H_2_SO_4_, and the plates were read at 492 nm in a Multiskan Plate Reader (MCC/340).

The results of the ELISA using synthetic peptides as antigens (EP) were compared with those obtained with the EIE-LVC Bio-Manguinhos kit, which is based on immunoenzymatic detection of canine visceral leishmaniasis. The EIE-LVC Bio-Manguinhos kit uses crude antigens and is currently recommended by the Brazilian Ministry of Health for the screening of seroreactive animals [18]. To do this comparison, the same serum samples were tested using both assays; the EIE-LVC kits were used according to the manufacturer's instructions and also calculating a different cut off, employing ROC curve and the same control serum samples already described.

### Statistical analysis

A cut off point for optimal sensitivity and specificity was determined using ROC analysis [19], and the area under the curve (AUC) was calculated to assess the performance of the tests. All of the statistical analyses were performed using GraphPad Prism™ (version 5.0) and MedCalc™ (version 7.3).

Agreement beyond chance between the tests was assessed using the Cohen Kappa (κ) coefficient [20] and interpreted according to the following scale: 0.00–0.20, negligible; 0.21–0.40, weak; 0.41–0.60, moderate; 0.61–0.80, good and 0.81–1.00, excellent [21]. The accuracy of each test was evaluated according to the AUC referent to the ROC curve and the Youden index J [22].

## Results

### Epitope identification, Spot synthesis and immunoassays with cellulose-bound peptides

ABCPred and BCPreds programs simultaneously predicted 191 distinct peptides. However, 11 peptides predicted by both programs were previously predicted by BepiPred [9]. All of these peptides (n = 360) were synthesized in cellulose membranes and submitted to immunoassays. Among these 360 peptides, there were 48 with RI ≥ 2, which are presented in Table S1.

All of the 48 selected peptides were synthesized onto new cellulose membranes and subjected to immunoassays with individual canine serum samples obtained from 5 uninfected dogs and 20 *L. infantum*-infected dogs. The pattern of recognition of the various serum samples was similar against the same peptide; furthermore, uninfected serum samples always showed lower reactivity compared to serum samples from infected animals. In this step, peptides that were reactive with pooled sera from *T. cruzi*-infected animals were excluded (data not shown).

Ten peptides were reactive against multiple individual canine serum samples (from 35% to 75% of samples) and also did not cross-react with pooled sera from *T. cruzi-*infected animals (presented in Table S2). Coincidently, these 10 peptides resulted from the simultaneous prediction by two programs (ABCPred and BCPreds). These peptides were then synthesized in a soluble form using solid phase technique to be used in ELISA tests.

### ELISA

The antigens (peptides) selected for ELISA testing were named as follows: PSLc1, PSLc2, PSLc3, PSLc4, PSLc5, PSLc6, PSLc7, PSLc8, PSLc9 and PSLc10. All of the peptides were mixed into a single solution (Mix10), which was used as another antigen in ELISA testing.

Peptide sensitivity and specificity were calculated using parasitological results as a gold standard. Most of the peptides were able to detect a large percentage of symptomatic and asymptomatic infected dogs, which was not observed with EIE-LVC kit. Diagnostic performances of the EP and EIE-LVC kit for canine serum samples are shown in Table 1. Based on the accuracy of the EP, those peptides with higher AUC and Youden index J values were selected. Thus, PSLc6, PSLc8 and PSLc10, as well as the Mix10, were tested again with a higher number of serum samples.

**Table 1:** Diagnostic performance of EP and EIE-LVC kit in serum samples from symptomatic and asymptomatic dogs.

Antigen/test	Se %	Sp %	AUC	Youden index J	Sym + (%)	Asym + (%)	Total+ (%)
**PSLc1**	72.50	75.00	0.754	0.475	17 (73.9%)	28 (71.7%)	45 (72.5%)
**PSLc2**	75.80	70.00	0.769	0.458	16 (69.5%)	31 (79.4%)	47 (75.8%)
**PSLc3**	74.10	70.00	0.778	0.441	22 (95.6%)	24 (61.5%)	46 (74.1%)
**PSLc4**	75.80	70.00	0.797	0.458	11 (47.8%)	36 (92.3%)	47 (75.8%)
**PSLc5**	70.96	55.00	0.642	0.259	16 (69.5%)	28 (71.7%)	44 (70.9%)
**PSLc6**	79.00	85.00	0.904	0.64	16 (69.5%)	33 (84.6%)	49 (79.0%)
**PSLc7**	85.40	80.00	0.863	0.654	19 (82.6%)	34 (87.1%)	53 (85.4%)
**PSLc8**	82.26	95.00	0.944	0.772	17 (73.9%)	34 (87.1%)	51 (82.2%)
**PSLc9**	79.00	75.00	0.847	0.540	12 (52.1%)	37 (94.8%)	49 (79.0%)
**PSLc10**	88.70	85.00	0.947	0.737	20 (86.9%)	35 (89.7%)	55 (88.7%)
**Mix10**	75.81	95.00	0.916	0.708	13 (56.5%)	34 (87.1%)	47 (75.8%)
**EIE-LVC kit** *	8.00	100.00	NA	0.08	5 (21.7%)	0 (0%)	5 (8.0%)

Samples from symptomatic (n = 23), asymptomatic (n = 39) *L. infantum-*infected dogs and healthy dogs (n = 20) were tested. Se: sensitivity; Sp: specificity; Sym: number of symptomatic dogs diagnose as positive; Asym: number of asymptomatic dogs diagnose as positive;

*cut off obtained according to the manufacturer.

**Figure 1:**
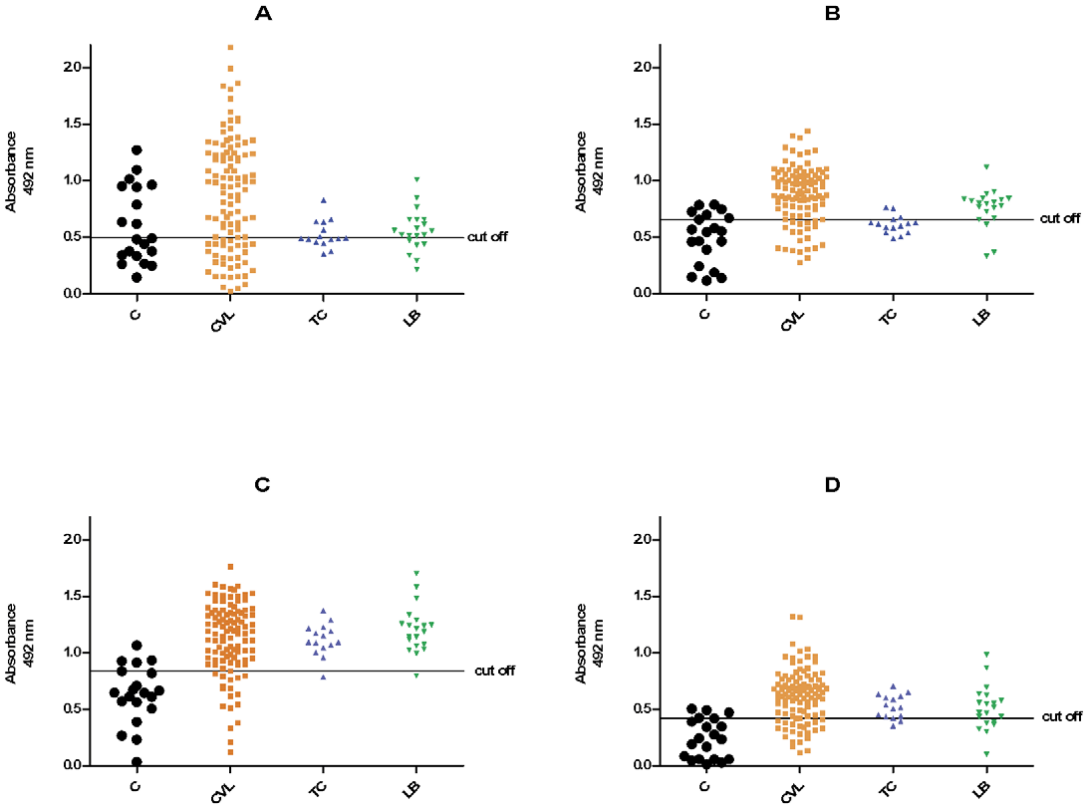
Comparison of ELISA reactivity of canine sera against PSLc6, PSLc8, PSLc10 and Mix10. ELISA was performed in different groups of dogs (C, control group, n = 20; CVL, *L. infantum* group, n = 107; TC, *T. cruzi* group, n = 15; LB, *L. braziliensis* group, n = 20) against PSLc6 (A), PSLc8 (B), PSLc10 (C) and Mix10 (D).

The next stage of testing was performed with 107 serum samples from *L. infantum*-infected dogs and the same 20 serum samples that were used as the negative controls in the previous assays. Additionally, 15 serum samples from *T. cruzi*-infected dogs and 20 from *L. braziliensis*-infected dogs were also tested. Figure 1 shows the results obtained with the three selected peptides and the Mix10 tested separately. Some cross-reactivity with *T. cruzi* and *L. braziliensis* occurred for all of the antigens, being cross-reactivity with *L. braziliensis* more frequent. For PSLc6, 40% of *T. cruzi* and 70% of *L. braziliensis*-infected serum samples were considered positive (Figure 1A). PSLc8 exhibited cross-reactivity with 26.6% of *T. cruzi* and 85% of *L. braziliensis*-infected serum samples (Figure 1B), whereas 93.3% of *T. cruzi* and 95.0% of *L. braziliensis*-infected samples reacted with PSLc10 (Figure 1C). Mix10 showed a similar pattern, with 80% of *T. cruzi*-infected serum samples and 70% of *L. braziliensis*-infected serum samples testing positive (Figure 1D).

**Figure 2:**
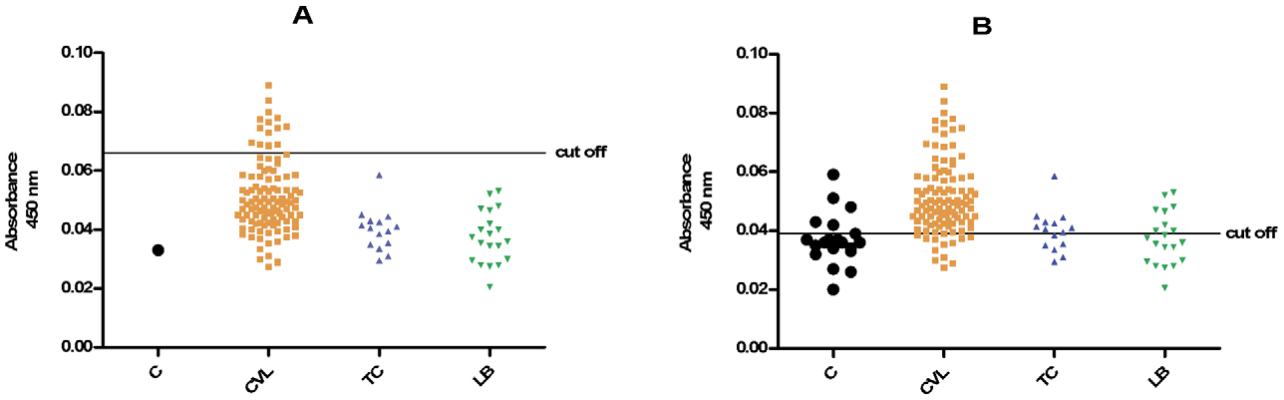
Comparison of ELISA reactivity of canine sera assessed by EIE-LVC kit. ELISA was performed in different groups of dogs (C, control group, n = 1, given by the kit; CVL, *L. infantum* group, n = 107; TC, *T. cruzi* group, n = 15; LB, *L. braziliensis* group, n = 20). In A, cut off was performed according to the manufacturer; in B, it was performed using ROC curve with serum samples from control group.

The same serum samples were tested using the kit recommended by the Brazilian Ministry of Health (EIE-LVC kit). The cut off value obtained as recommended by manufacturer (negative control absorbance multiplied by two) was very high, and was therefore outside the detection range for many of the infected dogs (Figure 2A). Furthermore, when ROC curve was applied using the previous described control serum samples, the cut off obtained was lower, which increased sensitivity, but cross-reactivity occurred with 53.3% of *T. cruzi* infected sera and with 40.0% of *L. braziliensis* infected sera (Figure 2B).

The sensitivity, specificity, AUC and Youden index J values were calculated for the investigated serological tests (Table 2). The sensitivity of the widely used EIE-LVC kit performed according to the manufacturer was 13.08%, but when ROC curve was applied to calculate cut off value, the sensitivity was 87.85%. EP showed sensitivities that ranged between 71.03% and 84.10%, depending upon the peptide utilized. The EP Mix10 presented the highest AUC value (0.902), showing a high accuracy [23], followed by the EP PSLc10 with an AUC value of 0.888. The Youden index J values for these two tests were also the highest, with values of 0.585 and 0.641, for the EP Mix10 and EP PSLc10, respectively. Specificity rates were determined for all uninfected dogs. The specificity of the kit was 100%, as a result of the high cut off value. In contrast, the specificity of EP ranged from 55% to 80%, depending on the peptide used.

**Table 2:** Diagnostic performance of EP and EIE-LVC kit in a larger panel of canine serum samples.

Serological test	Sensitivity in CVL group % (95% CI)	Specificity in control group % (95% CI)	AUC	Youden index J
**EP PSLc6**	71.03 (61.4–79.3)	55.0 (31.5–76.9)	0.661	0.260
**EP PSLc8**	81.3 (72.6–88.1)	70.0 (45.7–88.1)	0.876	0.513
**EP PSLc10**	84.1 (75.7–90.4)	80.0 (56.3–94.2)	0.888	0.641
**EP Mix10**	78.5 (69.5–85.8)	80.0 (56.3–94.2)	0.902	0.585
**EIE-LVC kit** *	13.08 (7.3–20.9)	100.0 (2.5–100.0)	NA	0.130
**EIE-LVC kit** #	87.85 (80.12– 93.3)	75.0 (50.9–91.3)	0.848	0.620

Samples from *L. infantum-*infected dogs (n = 107) and healthy dogs (n = 20) were tested. CI: confidence interval; AUC: area under the curve; NA: not applicable;

*cut off obtained according to the manufacturer;

#cut off obtained by ROC curve using 20 control serum samples.

**Figure 3:**
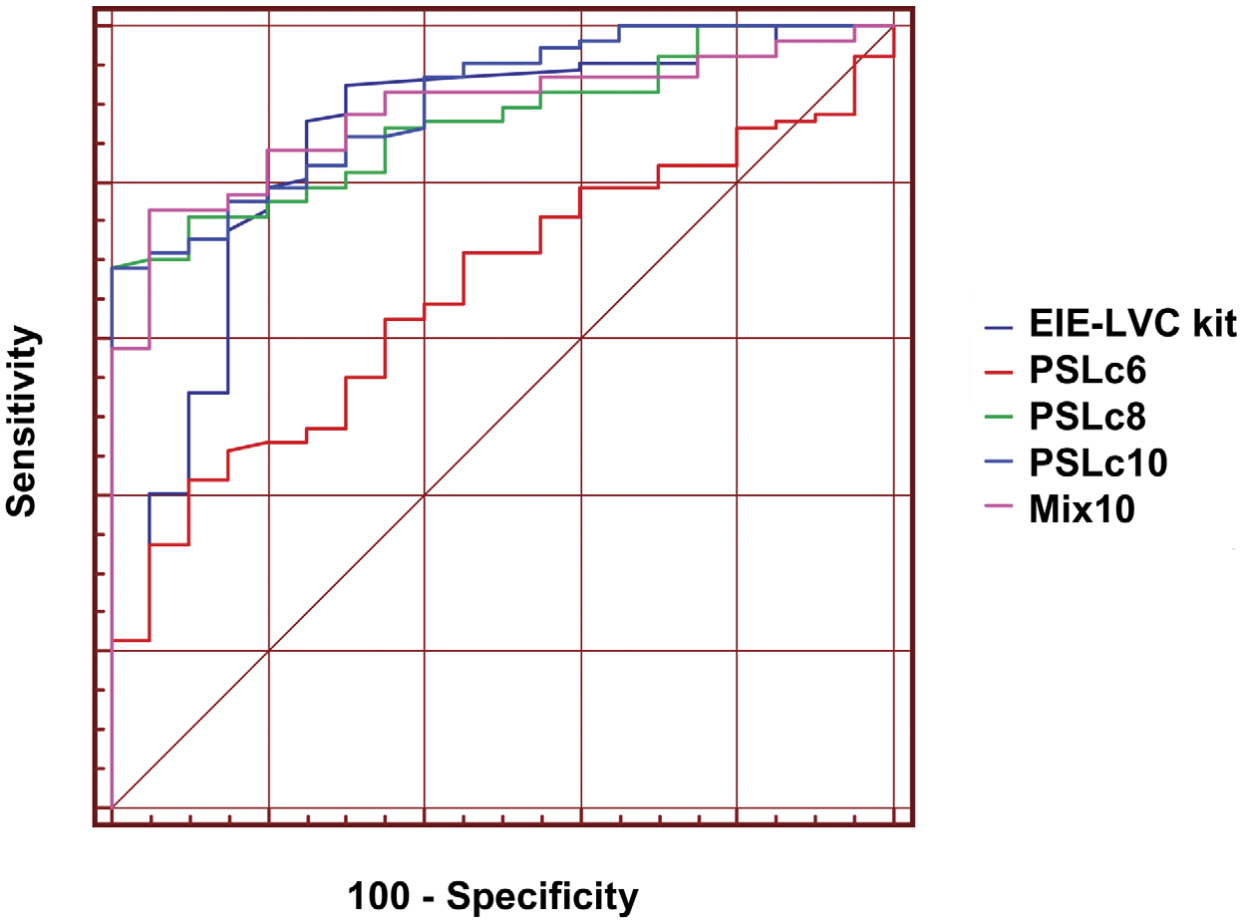
Comparison of ROC curves obtained from all the tests. The curves were used to determine ELISA cut off, sensitivity, specificity and AUC.

When the ROC curves obtained from all of the investigated tests with synthetic peptides were combined, it was possible to observe that EP PSLc6 had the lowest AUC value, showing the most ineffective performance of all of the tests. In contrast, the other tests presented similar performances, showing overlapping ROC curves (Figure 3).

A good agreement beyond chance (κ index) ranging from 0.402 to 0.751 was obtained when the results from each peptide (PSLc6, PSLc8, PSLc10) and Mix10 were cross-tabulated (Table 3). Among these results, the κ index value for PSLc6 and Mix10 was the lowest (0.402), showing weak agreement. The correlation between PSLc8 and Mix10 had the highest κ index value (0.751), showing a good agreement. When the EIE-LVC kit performed according to the manufacturer was cross-tabulated with the synthetic peptides, the agreement was very poor. The index ranged between 0.031 (compared to Mix10) to 0.064 (compared to PSLc8). All of these correlations indicate a negligible agreement between these tests. When EIE-LVC kit performed using ROC curve and 20 control serum samples was cross-tabulated with the synthetic peptides, the agreement was weak. It ranged from 0.213 (compared to PSLc6) to 0.406 (compared to PSLc10).

**Table 3:** Kappa index (κ) between paired results of diagnostic tests.

Serological test		Serological test														
		EIE-LVC kit *			EP PSLc6			EP PSLc8			EP PSLc 10			EP Mix10		
		P (+)	N (−)	T	P (+)	N (−)	T	P (+)	N (−)	T	P(+)	N(−)	T	P(+)	N(−)	T
**EIE-LVC kit** #	**P(+)**	14	87	101	73	28	101	81	20	101	84	17	101	76	25	101
	**N(-)**	0	26	26	12	14	26	12	14	26	10	16	26	12	14	26
	**T**	14	113	127	85	42	127	93	34	127	94	33	127	88	39	127
κ **index (95% CI)**		0.062 (-0.049–0.172)			0.213 (0.011–0.415)			0.306 (0.097–0.514)			0.406 (0.208–0.605)			0.245 (0.041–0.450)		
**EP Mix10**	**P(+)**	11	77	88	70	18	88	84	4	88	81	7	88	-		
	**N(-)**	3	36	39	15	24	39	9	30	39	13	26	39			
	**T**	14	113	127	85	42	127	93	34	127	94	33	127			
κ **index (95% CI)**		0.031 (-0.098–0.161)			0.402 (0.227–0.578)			0.751 (0.622–0.878)			0.613 (0.458–0.769)					
**EP PSLc 10**	**P(+)**	13	81	94	75	19	94	85	9	94	-			-		
	**N(-)**	1	32	33	10	23	33	8	25	33						
	**T**	14	113	127	85	42	127	93	34	127						
κ **index (95% CI)**		0.060 (-0.061–0.182)			0.455 (0.280–0.629)			0.655 (0.503–0.808)								
**EP PSLc8**	**P(+)**	13	80	93	75	18	93	-			-			-		
	**N(-)**	1	33	34	10	24	34									
	**T**	14	113	127	85	42	127									
κ **index (95% CI)**		0.064 (-0.059–0.186)			0.477 (0.306–0.648)											
**EP PSLc6**	**P(+)**	11	74	85	-			-			-			-		
	**N(-)**	3	39	42												
	**T**	14	113	127												
κ **index (95% CI)**		0.041 (-0.094–0.175)														

Samples from *L. infantum-*infected dogs (n = 107) and healthy dogs (n = 20) were tested. P: positive; N: negative; T: total; CI: confidence interval;

*cut off obtained according to the manufacturer;

#cut off obtained by ROC curve using 20 control serum samples.

## Discussion

Despite the efforts to search for new diagnostic methods, a method with satisfactory CVL diagnosis efficiency is not yet available. The use of accurate methods that are easy use in the field and are cheap is crucial for diagnosis and consequently, for disease control. Therefore, the identification of new antigens is an important research area for VL disease control. In this work, new antigens for CVL serological diagnosis were investigated using bioinformatic tools and successive screenings with immunoassays.

Using bioinformatics, 360 epitopes were predicted from 47 immunogenic proteins that were identified by bidimensional electrophoresis and Western blot [9]. B cell prediction showed great efficiency because, in the immunoassays, the majority of the cellulose-bound peptides were immunogenic. Of the 48 peptides that could be used to differentiate infected and uninfected dogs, we selected the ten most specific ones.

The ten best peptides resulted from the prediction of two programs simultaneously (BCPreds and ABCPred), and none resulted from using BepiPred considering 2 as a minimum score. However, we observed that 60% of the ten best peptides were also predicted as epitopes based on BepiPred's default score (0.35). These results suggest that using the default score of prediction programs associated with the overlapping predictions by more than one program can be better than using a single type of prediction. Several authors have already shown that combined T cell epitopes generated using consensus predictions are believed to be more accurate [24], [25]. It is important to mention that ABCPred and BCPreds approaches are similar (machine learning methods), whereas the BepiPred approach is based on propensity scale methods. This difference could potentially explain the differences between the epitope predictions made by BepiPred alone compared to those made by the ABCPreds and BCPreds programs together.

After the immunoassays in cellulose membranes, the ten peptides that reacted with multiple tested serum samples were synthesized using Fmoc technique. These peptides showed good reactivity as antigens and were able to recognize specific antibodies in ELISA. The performance observed for the new antigens presented in this work (maximum of sensitivity and specificity of approximately 84.1% and 80.0% respectively) is compatible with the performance of many antigens, mainly recombinant proteins, that have been developed in recent years for CVL diagnosis [26]-[28] with the advantage of being cheaper and faster to produce.

However, there are other ELISA methods described for CVL that have greater sensitivity or specificity compared to EP. A study conducted in Brazil investigated sera from negative controls (n = 30) and from *L. infantum-*infected dogs (n = 60) in a fucose-mannose ligand based ELISA. Using ROC curve, the sensitivity was 90% and specificity was 93.3% in oligosymptomatic dogs [29]. Another Brazilian study analyzed 209 sera samples from *L. infantum-*infected dogs in a recombinant cysteine proteinase based ELISA. The cut off value was obtained by adding two standard deviation values to the mean absorbance of 22 sera samples from healthy dogs. It provided values of 98% and 96% from sensitivity and specificity respectively [30]. In Europe, some researchers investigated soluble antigens derived from promastigote or amastigote-like stages of *L. infantum* in ELISA. The cut off value was the arithmetic mean plus 3 standard deviations of 48 negative controls. When a group of 47 *L. infantum*-infected dogs was tested, sensitivity varied from 94.1 to 100%, and specificity varied from 96 to 100% [31]. All of these findings show that much has been done to improve CVL diagnosis, but the results are similar. Higher values of sensitivity and specificity are motivations for our antigen improvement.

We compared the results obtained in EP with those obtained with the widely used kit recommended by the Brazilian Ministry of Health, the EIE-LVC kit. Evaluation of EIE-LVC kit, used as recommended by manufacturer, revealed a sensitivity of 13.08% and a specificity of 100%. This low sensitivity could be explained by the high cut off value, which missed many infected dogs. However, following this manufacturer's instruction, test accuracy is related to the composition of only one negative control provided by the kit. Then, we used also 20 serum samples from known uninfected dogs, and the cut off values were obtained using ROC curves. This way, it was possible to obtain sensitivity of 87.8% and specificity of 75.0%. The performance of this kit, when evaluated by other authors is variable. Values of sensitivity of approximately 87.5% and specificity of approximately 100% were obtained elsewhere; 15 false negative results were reported, being 11 in asymptomatic dogs, in a group of 120 samples. Therefore, the prevalence of CVL was underestimated [32]. However, using the same kit, another study showed a sensitivity of 72% and a specificity of 87.5%. These authors indicated the use of EIE-LVC kit in parallel with another kit produced by Bio-Manguinhos, which employed indirect immunofluorescence, to minimize the number of false negatives [33].

Previous data have shown that serologic test performance in CVL depends on infection status [34], [35] and an important limitation in CVL control programs is the inability to identify asymptomatic dogs because classic diagnostic tests are insufficiently sensitive [36]. Thus, a sensitive and specific antigen for the detection of asymptomatic dogs would be highly desirable because it would allow for effective control intervention in areas where CVL occurs. In our tests, the EIE-LVC kit was not able to detect any of the 39 serum samples from asymptomatic dogs, while the new antigens exhibited strong reactivity to the tested sera. For example, in symptomatic dogs, positive reactions reached up to 95.6% and in asymptomatic dogs, reached up to 94.8%, depending upon the used peptide.

Among the tested peptides, PSLc6, PSLc8, PSLc10 and Mix10 showed the highest accuracies when tested with serum samples from animals with defined clinical statuses. Thus, they were selected to be tested with more serum samples. Then, we observed that EP Mix10 showed the best performance, with an AUC of 0.902, characterizing the test as highly accurate. EP PSLc8 and EP PSLc10 had AUC values that characterized these tests as moderately accurate; only PSLc6 generated a test with low accuracy. The highest value of sensitivity (84.1%) also was obtained when PSLc10 was used as antigen. In a canine epidemiological screening, a test with high sensitivity is desirable.

In addition, all of the EP assays showed a good agreement according to the κ index, when they were cross-tabulated. However, when the EP tests were cross-tabulated with the EIE-LVC kit, the agreement was poor in both situations: as performed according to the manufacturer, it was negligible and as performed with our control serum samples, it was weak. It could be explained mainly by the different antigens employed in the tests. The EP appeared to be more sensitive, once it uses synthetic peptide while the EIE-LVC kit employs crude antigen. Thus, even using the ROC curve, the agreement still remained weak between EIE-LVC kit and EP tests.

The occurrence of cross-reactivity with *T. cruzi* and *L. braziliensis* in EP were observed with all synthetics antigens. These results corroborate other researchers' findings concerning to new antigens in CVL diagnosis [26], [27], [37], [38]. In all of them, some cross-reactivity with these parasites occurred.

Although there has been speculation on the role of dogs in the zoonotic cycle of tegumentary leishmaniasis (TL) caused by *L. braziliensis,* only circumstantial evidence supports this hypothesis [39]. Indeed, the primary reservoirs of *L. braziliensis* are small mammals, particularly wild rodents [40], making the importance of this kind of cross-reactivity in the diagnosis of CVL controversial. Therefore, it could be considered that TL infected dogs have low antibodies levels similar to human TL infections. Some authors described the immune response in human TL infection as predominantly cellular, with low levels of circulating antibodies [41].

Importantly, canine infection with *L. braziliensis* is associated with rural areas. Our test has been developed with the intention of being used in urban surveys, as a diagnostic tool in the control of urban LV. Besides, the prevalence rates of *L braziliensis* in dogs are low (3.1%), when parasitological examination was performed in Brazil [42]. About the prevalence of *T. cruzi* in dogs, few data have been published. In a study conducted with 244 dogs in nine municipalities in Paraná state, Brazil, no dogs were found to be infected with *T. cruzi* [43]. For this reason, it is difficult to establish the real importance of cross-reactivity with these trypanosomatids in EP tests.

The existence of false positives related to these tripanossomatids (*L. braziliensis* and *T. cruzi*) raises the suspicion of cross reaction with another parasites with higher prevalence in urban dogs, such as *Ehrlichia canis* and *Babesia canis*. Further investigation will be necessary in order to characterize the occurrence of potential cross reactions in EP.

Recent studies have evaluated multiple-epitope chimeric antigens as diagnostic markers for the serodiagnosis of CVL [26], [44], which represents an interesting approach to our peptides. The development of an immunochromatographic strip would also be desirable, as it is a common approach in the diagnosis of CVL (rK39 strips) and other pathologies [45]–[47]. It would be interesting to combine multiple peptides to improve the accuracy, as CHEMBIO has done with the DPP™ immunochromatographic test that employs recombinant antigens K39 and K26 to diagnose CVL. It is feasible to use in the same strip test an antigen with good sensitivity and another with good specificity. Taken together, to improve the diagnostic specificity preventing the unnecessary culling of dogs, some alternatives employing the synthetic peptides should be further investigated. For example, structural changes in the antigens, such as the production of conjugated peptides would be an interesting approach in order to increase both sensitivity and specificity.

In conclusion, we have designed new synthetic peptides for the improved serodiagnosis of CVL. The synthetic peptides named PSLc6, PSLc8, PSLc10 and Mix10 afforded high accuracy in detecting CVL cases and are faster and cheaper to produce. Our findings indicate that synthetic peptides will be useful for serodiagnosis and allow for the detection of asymptomatic dogs. The development of an immunochromatographic test using these peptides would be a valuable tool for the rapid diagnosis of CVL, an important issue for the control of this neglected disease in endemic areas.

## Supporting Information

Table S1 **Peptides with relative intensity (RI) equal or greater than 2.**
(XLS)

Table S2 **Reactivity of serum samples to 48 different peptides.**
(XLS)
